# Acute retinal pigment epitheliitis during treatment of hyperprolactinaemia

**DOI:** 10.1186/s12886-024-03366-0

**Published:** 2024-03-01

**Authors:** Małgorzata Kowalik-Jagodzińska, Karolina Czajor, Anna Turno-Kręcicka

**Affiliations:** 1 Department of Ophthalmology, Wrocław Medical University, Wroclaw, Poland; 2 Clinic of Ophthalmology, University Teaching Hospital in Wroclaw, Wroclaw, Poland

**Keywords:** Case report, Krill disease, Hyperprolactinaemia, Bromocriptine, Cabergoline

## Abstract

**Background:**

Acute retinal pigment epitheliitis (ARPE) is a rare, idiopathic and self-limiting disease. The article aims to present ARPE in a patient using D2 dopamine receptor agonists for the treatment of hyperprolactinemia.

**Case presentation:**

A 28-year-old female during hyperprolactinaemia treatment suffered from a dyschromatopsia and a central visual field defect in the left eye. She noticed a deterioration of vision and discontinued the cabergoline administration. The woman had not been diagnosed with other chronic conditions and exhibited no symptoms of infection. Upon admission, the patient was subjected to a test for COVID-19, which was negative.

The ophthalmological examination revealed a decrease in visual acuity to distance in the left eye, which amounted to 18/20 on the Snellen chart. A central scotoma was noted on the Amsler chart and a loss of pigment epithelium was visible on the fundus of the left eye. Fluorescein angiography showed a discrete window defect in the left one, with no signs of leakage. Optical coherence tomography (OCT) scans of the maculae revealed a characteristic change in the photoreceptor layer and retinal pigment epithelium (RPE) in the fovea in the left eye. The electrophysiological tests revealed decreased function of cells in macular region.

A magnetic resonance imaging (MRI) of the head and orbits demonstrated an asymmetric pituitary gland without chiasm compression and discrete signal enhancement from the left optic nerve.

The patient underwent observation during hospitalisation. She reported improved colour vision and a decreased scotoma in the centre of her visual field.

In regular outpatient follow-ups, successive improvements in visual acuity, as well as a decreased RPE damage and outer photoreceptor layer loss during an OCT test were observed.

**Conclusions:**

A case of ARPE is reported in a patient taking medications for hyperprolactinemia. The role of dopamine receptor antagonists in the photoreceptor function and causation of ARPE needs further evaluation.

## Introduction

Acute retinal pigment epitheliitis is a retinal condition, usually unilateral, that most often affects young people. The disease was first described by Krill in 1972 [1]⁠. In general, it is not associated with other conditions, does not require any therapeutic intervention and resolves spontaneously, although the literature includes reports of recurrent episodes of inflammation [2, 3]. Due to a small number of cases, an objective assessment of the causes of this disorder is significantly inhibited, although a viral basis has been suggested [4]. Based on the analysis of imaging tests, i.e. fluorescein angiography, indocyanine angiography and optical coherence tomography, it was discovered that the destruction involves the inner and outer segments of photoreceptors and pigment epithelium within the fovea and begins directly at the junction between the outer segments of photoreceptors and pigment epithelial cells [5]. The OCT cross-section of the retina showed a characteristic hyperreflective dome-shaped lesion extending from the RPE through the outer and inner layers of the photoreceptors with a break in the continuity of these layers [5] – in some cases, reaching the outer nuclear layer [6]. Based on the analysis of the correlation of changes observed in OCT images with the return of baseline visual acuity during the course of the disease, conducted by Iu et al., it was noted that disruption of the ellipsoid and interdigitation zones occurs in each case, and Improvement in visual acuity takes place when the interdigitation zone is restored, which happens between 3 and 6 months after the onset of the disease [7, 8]. In cases where the lesions reach the outer nuclear layer, visual acuity is weakest and may not completely recover at a 1-year follow-up [9].

## Case presentation

A 28-year-old patient was electively admitted to the Department of Ophthalmology of the University Teaching Hospital in Wrocław for about 3 weeks in order to diagnose a left eye vision disorder. The patient suffered from a central visual field defect and colour vision abnormality- blue became green, yellow became beige and red became grey. The patient’s medical history indicated that she visited a gynaecologist around 2 months before the onset of the presented symptoms due to a libido disorder. Hormone tests were performed, which showed elevated serum prolactin levels. The patient’s treatment began with bromocriptine (Bromergon; Sandoz, Domaniewska 50C Warszawa) administered for 6 weeks in a dose of 0,625 mg per day. Because of incomplete success of treatment, the drug was changed into cabergoline (Dostinex; PFIZER EUROPE MA EEIG Ramsgate Road Sandwich CT13 9NJ United Kingdom; 0,5 mg per week on Saturdays). The drugs belong to the D2 dopaminergic receptor agonist group. The patient discontinued the cabergoline administration after the sixth dosage due to the observed central scotoma in her left eye. Seeing no spontaneous improvement, the woman went to the hospital emergency department. A computer tomography (CT) scan of the head and orbits, along with ophthalmological and neurological examinations, were performed. There were no deviations in CT examination as well as pathological signs during neurological assessment.

The patient was referred to the ophthalmology department for diagnosis. Due to the coronavirus pandemic, a PCR nasopharyngeal smear test for SARS-CoV2, which gave a negative result, was conducted prior to admission. The woman did not exhibit symptoms of infection. She had taken the COVID-19 vaccine (Comirnaty; Pfizer/BioNTech Manufacturing) 5 days before the admission, which was around 2 weeks after the appearance of visual symptoms, so that the possible vaccination influence was precluded [10].

**Fig. 1:**
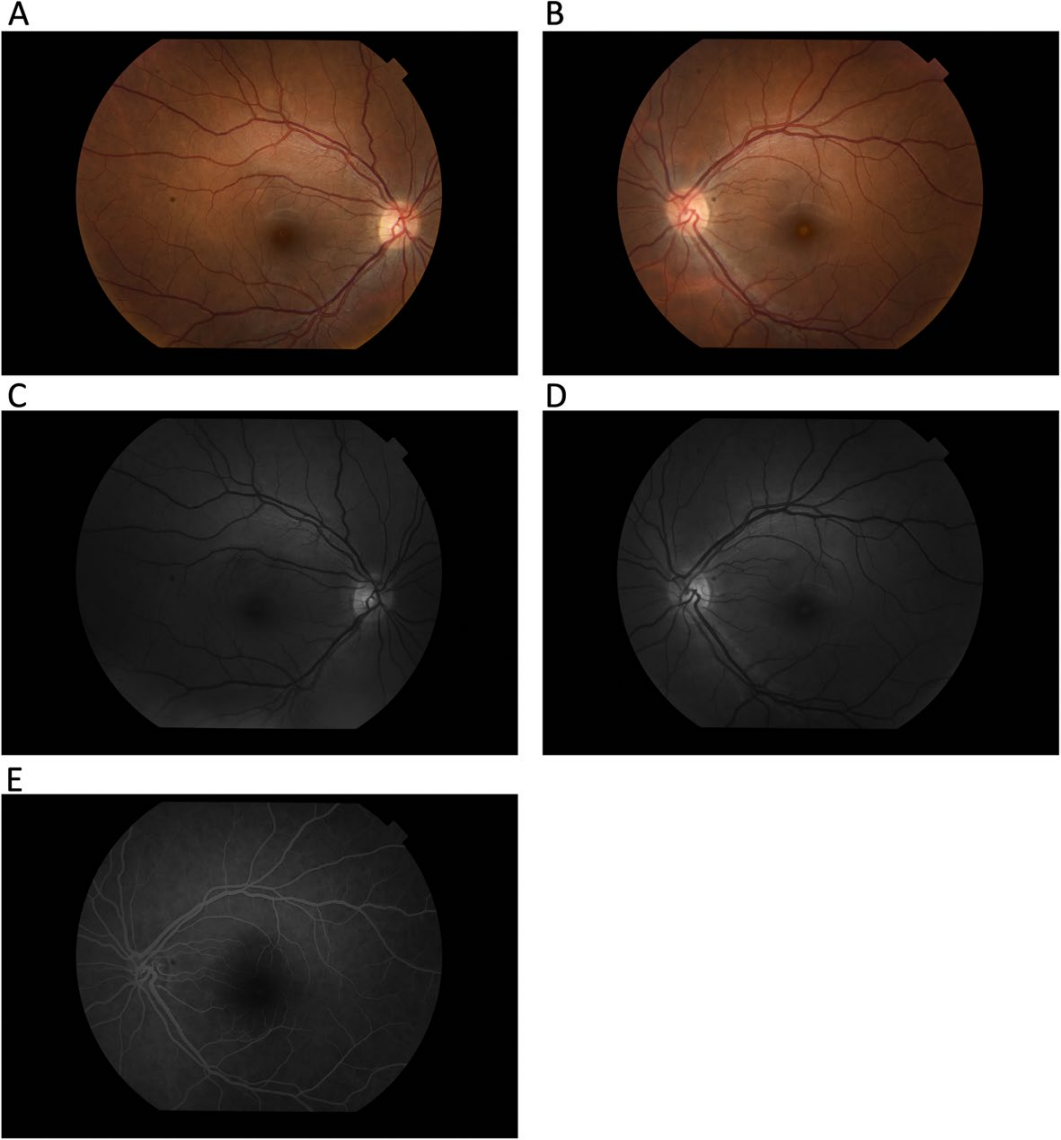
**A** Color fundus image of the right eye, **B** Color fundus image of the left eye, **C** Red free image of the right eye, **D** Red free image of the left eye, **E** Fluorescein angiography- left eye (Time after fluorescein administration: 1:05.6) Figure shows retinal pigment epithelium defect (ring-shaped in the right eye and round in the left eye without dye leakage during angiographic examination of the left eye

The physical examination upon admission showed a decrease in visual acuity to distance in the left eye, which measured 18/20 on the Snellen chart, a central scotoma in her left eye during the Amsler test, as well as a loss of pigment epithelium visible on the fundus of the left eye (Fig. 1b). The right eye demonstrated no abnormalities in the aforementioned ophthalmological tests. The anterior segments of both eyes during a slit-lamp examination did not exhibit inflammatory features, and the optical media remained transparent.

The visual field examinations conducted using the Carl Zeiss Humphrey Field Analyser included several perimetry tests in different algorithms – the neurological test, the SITA-fast 24-2 test, and the macular degeneration test in algorithm 10.2. No absolute scotomata were observed during the aforementioned examinations.

The fluorescein angiography performed with a Topcon TRC 50 DX retinal camera (IMAGEnet I-base Version 3.25.0) revealed a circular lesion in the macular region of the left eye. No dye leakage in either phase or areas of hypoperfusion was shown (Fig. 1E).

**Fig. 2:**
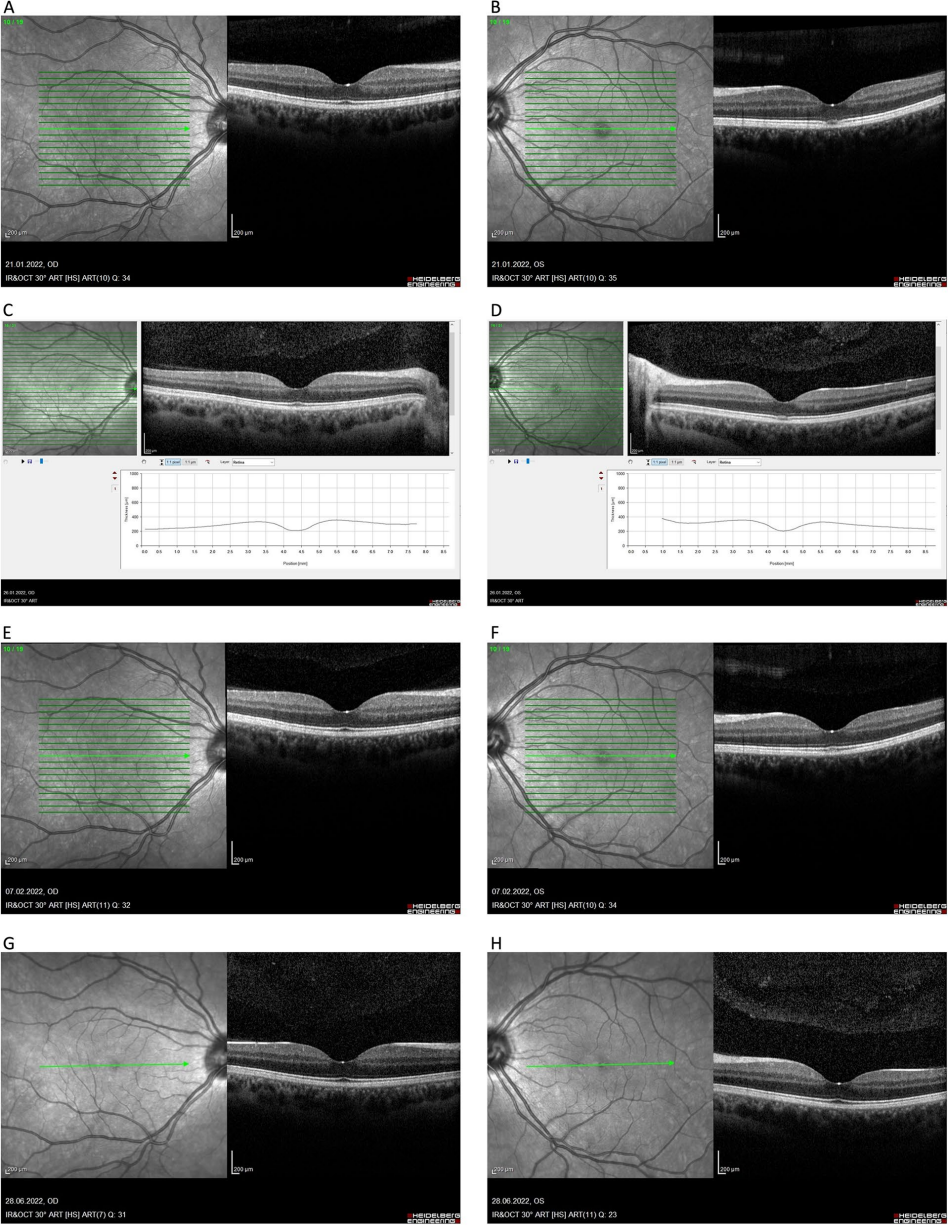
Spectral- domainOCT scans (OCT Spectralis, Heidelberg) showed presence and evolution of retinal pigment epithelium alterations in acute phase of disease (**A**-**F**) and after 6 months (**G**, **H**). Visual acuity was 20/20 on **F**

OCT scans of the maculae performed using the Heidelberg Eye Explorer Version 1.12.1.0 (SPECTRALIS software V 7.0.4) showed a characteristic defect in the outer portions of the photoreceptors and the RPE in the left eye fovea, as well as a hyperreflective lesion involving the photoreceptor layers (Fig. 2). In the right eye, the OCT images presented no abnormalities; the foveal outline and stratified structure were preserved. No features of oedema were observed. The characteristic image indicated ARPE [11].

**Fig. 3:**
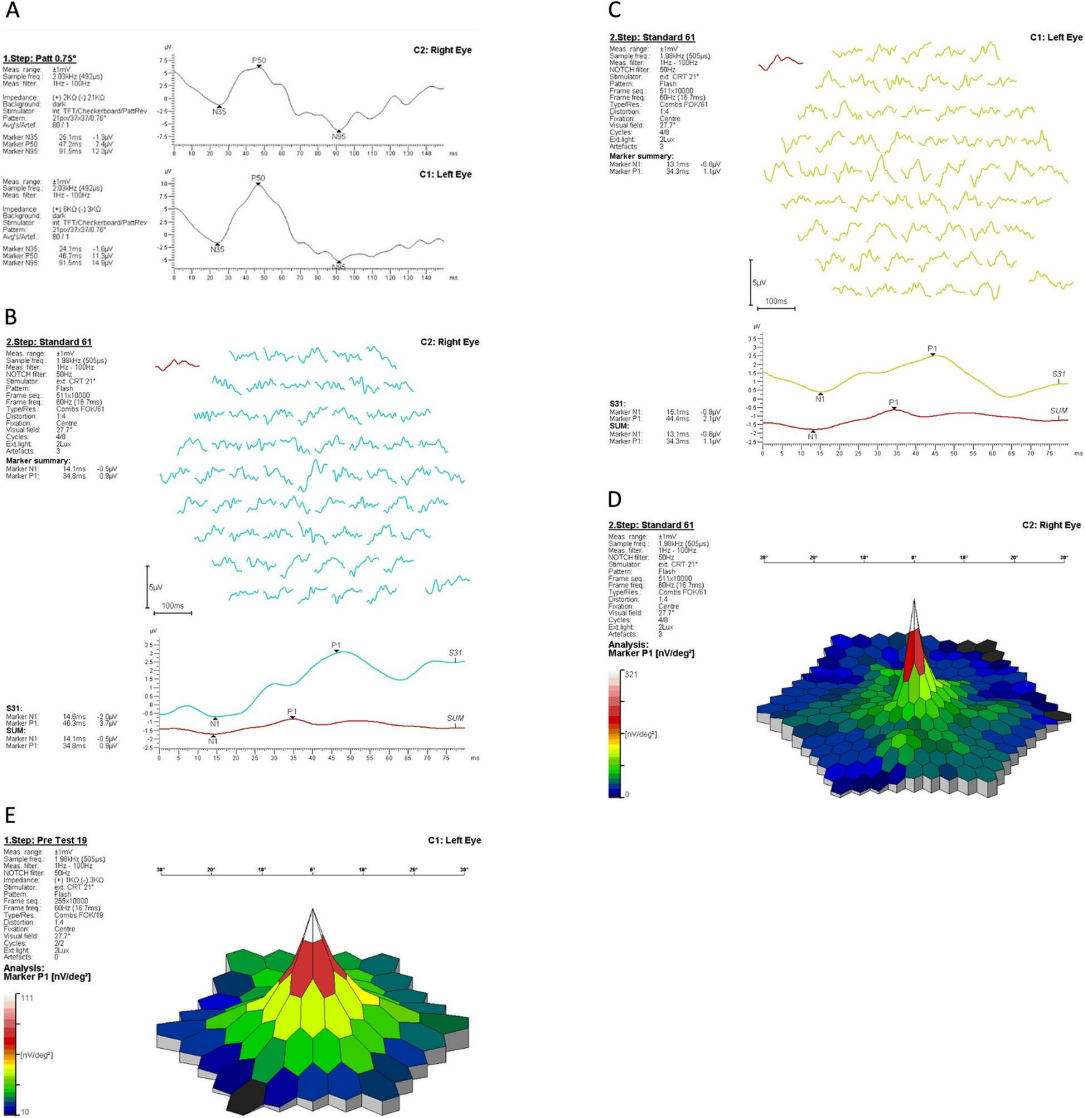
Electrophysiology. **A** ERG schowed reduction in amplitude of the P50 component in right eye (acute phase of disease); **B**-**E** mfERG showed decrease of response density in middle circles- especially in left eye. **B** Right eye and **C** left eye- waves; **D** right eye and **E** left eye 3-dimentional distribution model

Electrophysiological tests confirmed reduction of electrical activity of cells in macular region. The results of the pattern electroretinography (pERG)– showed decline P50 wave amplitude (represents function of outer retinal cells in macular region) of the right eye measured around 50% relative to the left eye (Fig. 3A). The response densities of multifocal-ERG (created mostly by activity of cones and bipolar cells) were also decreased, especially in left eye (EP 1000 PC TOMEY, Software version 3.2.0, Fig. 3B-E). That is why it is possible that ARPE in a described case was bilateral but on the right side such early changes were not visible for the patient and not detectable by imaging techniques.

Blood tests examining the function of the pituitary, thyroid and adrenal cortex were also performed to expand the diagnosis of causes of hyperprolactinaemia. The results were: Total beta-HCG < 1,2 mIU/ml (normal range for non-pregnant women 0–5,0 IU/l), TSH 1.25 μIU/ml (0.35–4.94), testosterone level 0.29 ng/mL (0.14–0.53), Cortisol 12.4 μg/dl (3.7–19.4 till 10 am- sample collected at 08:27 am), androstendione 1.15 ng/ml (0.3–3.3), DHEA-S 296.3 μg/dL (95.8–511.7), FSH 2.96 mIU/mL (follicle faze 3.03–8.08, peek in the middle of period 2.55–16.69, luteal faze 1.38–5.47, after menopause 26.72–133.41), LH 1.76 mIU/mL (follicle faze 1.8–11.78, peek in the middle of period 7.59–89.08, luteal faze 0.56–14.0, after menopause 5.16–61.99), prolactin 10.76 ng/mL (5.18–26.53; in patient’s documentation the level of prolactin measured circa 1 and a half a month ago was 38,79 ng/ml and norms for the laboratory were 4,79–23,30). Moreover, slightly noticeable leukocytosis: 10.11 *3/μL (4–10) was detected and normal function of kidneys and liver (creatinine 0.73 mg/dl (0.55–1.02, EGFR 100.0, urea 26.0 mg/dl 17–43, ALAT 14 and the normal range 0.35).

**Fig. 4:**
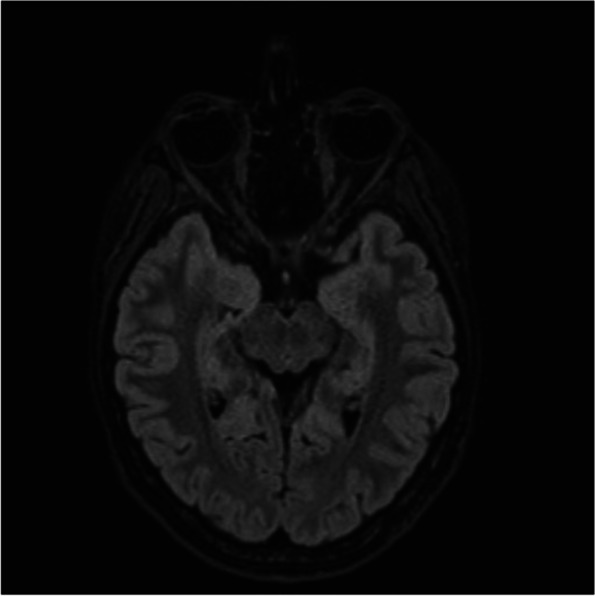
MRI image of the head and orbits revealing asymmetry of the pituitary gland structure without signs of compression on the optic chiasm

An MR of the head and orbits on a 1.5 T instrument revealed an asymmetrical structure of the pituitary gland (the posterior lobe of the gland is located on the left side of the Turkish saddle and the pituitary stalk is slightly displaced to the right side) but without features of glandular proliferation and pressure on the optic nerve chiasm (Fig. 4).

The patient did not undergo any treatment, yet she reported day-by-day improvement in colour vision and a decrease in the scotoma in the centre of her visual field what was corroborated with diminishing lesion in OCT scans (Fig. 2G, H).

Visual acuity returned to 20/20 after 1 month. The central scotoma and colour vision disorder disappeared at the same time. After nearly 6 months, no pathology was revealed in the OCT images of the maculae.

In addition, a multifocal ERG test was performed, which showed unstable fixation of the left eye, indicating a macular pathology of the left eye as the cause of the reported symptoms.

## Discussion

Acute retinal pigment epitheliitis can result from the toxic or immunogenic effects of drugs [12]. In the presented case, drugs in the form of dopamine D2 receptor agonists would be expected to cause changes consistent with ARPE due to their direct side effects on the function of photosensitive retinal cells.

Photoreceptors are cells that are sensitive to diurnal changes in light intensity, i.e. their functioning is dependent on the so-called daily biological clock. The function of retinal cells is based on signals from the outside world, that is, variations in light intensity throughout the day and internal signalling involving an alternating preponderance in the release of neuromodulators [13]. Dopamine is a transmitter that plays a key role in the proper control of diurnal rhythm and the associated shift in the activity of retinal ganglion cells [14]. The mediator influences two types of dopamine receptors, modulating the photoreceptor response and the intensity of the visual cascade response by increasing the concentration of cAMP (D1 receptors) or decreasing its concentration (by means of D2-type receptors), as well as affecting melatonin synthesis [15]. Dopaminergic receptors are found in the outer segments of photoreceptors, as confirmed in the tests [16]. The injection of dopamine or dopaminergic agonists into the area of the outer segments of photosensitive cells resulted in a reduction in their sensitivity to light. Under bright light conditions, dopamine synthesis and release increase to stimulate D2 receptors, which leads to an inhibition of gap junctions between cones and rods [13]. At night, the situation is reversed, resulting in an increase in the formation of the aforementioned connections and decreasing the signal-to-noise ratio, which is relevant for recognising shapes and objects in the dark.

The standard procedure in the case of hyperprolactinaemia involves the use of dopamine D2 receptor agonists [16] if elevated prolactin levels cause symptoms. In the case of premenopausal women, elevated prolactin levels may manifest themselves only as libido disorders, menstrual disorders or problems in becoming pregnant [17]. In cases where no symptoms are present, treatment is not necessary. The use of drugs aims to reduce the production and secretion of prolactin – some preparations (e.g. cabergoline) also reduce the volume of prolactinomas [18]. Comparing the duration of action of the substances, bromocriptine is usually administered once or twice a day, while cabergoline once or twice a week [18], which causes cabergoline to be better tolerated and often applied as a first-line drug in patients with prolactinomas [17, 19, 20].

The mfERG examination was used in the study due to its high sensitivity. For instance, the examination is recommended during screening for chloroquine and hydroxychloroquine retinopathy because alternations could be revealed before the visible changes on the fundus [21]. According to our case, we suppose that the disorder was bilateral with early stage of ARPE in the right eye (invisible in fundus examination and OCT). There is no similar case of ARPE with mfERG and OCT findings in literature.

Actually, it is discussed, if ARPE is a disease itself or the published cases are rather examples of the other, better known, disorders, such us multiple evanescent white dot syndrome (MEWDS), pachychoroid diseases or acute macular neuroretinopathy (AMN) after light damage [22, 23]. According to our case the patient denied a possibility of light damage but we could not exclude two other propositions. As it was mentioned in the cited position in our management autofluorescence (FAF) or indicyanine green (ICG) angiography are missing. Nevertheless, the background of known rare visual side effects of cabergoline administration described in Summary of Product Characteristics is probably discovered.

## Conclusion

During the diagnosis of the visual disturbances described by the patient, retrobulbar neuritis of the left eye, the influence of infectious factors [24] on the functioning of photoreceptors and the mechanism of their exfoliation through pigment epithelial cellswere taken into account. Given the abnormalities in multifocal electroretinography, which suggest primary pathology within the macular photoreceptors, optic neuritis appears to be unlikely in this case. Additionally, the characteristic morphology and OCT image findings indicates ARPE as the probable disorder in this case [11, 25]. Moreover, when subjective symptoms were present, there were no objective deviations, such as the presence of a relative afferent pupillary defect, colour vision abnormalities in the red-green axis or deviations in perimetry. The mentioned case of ARPE is hypothesised to be functional and structural dopamine-dependent condition due to spontaneous disappearance of symptoms in relation to discontinuation of hyperprolactynaemia treatment.

## Data Availability

All data are available upon request to the corresponding author.
